# Boredom, loneliness and modern type depression in a cohort of Italian university students

**DOI:** 10.1192/j.eurpsy.2022.2245

**Published:** 2022-09-01

**Authors:** L. Orsolini, S. Bellagamba, G. Longo, S. Tempia Valenta, V. Salvi, U. Volpe

**Affiliations:** 1 Unit of Clinical Psychiatric, Polytechnic University of Marche, Ancona, Italy, Department Of Neurosciences/dimsc, Ancona, Italy; 2 Unit of Clinical Psychiatry, Department Of Neurosciences/dimsc, Polytechnic University Of Marche, Ancona, Italy; 3 Polytechnic University of Marche, Department Of Clinical Neurosciences/dimsc, School Of Medicine, Unit Of Psychiatry, Ancona, Italy; 4 Unit of Clinical Psychiatry, Polytechnic University of Marche, Ancona, Italy, Department Of Neurosciences/dimsc, Ancona, Italy

**Keywords:** Modern Type Depression, Hikikomori

## Abstract

**Introduction:**

COVID-19-related physical isolation, fear and anxiety determined de novo mental illnesses, by potentially facilitating the emergence of Hikikomori traits (i.e., a severe social withdrawal condition).

**Objectives:**

The present study aims at screening a cohort of university students for the Hikikomori traits and assessing a set of psychopathological determinants associated with Hikikomori, particularly boredom and loneliness dimensions.

**Methods:**

A cross-sectional web-based survey was carried out by administering Hikikomori Questionnaire (HQ-11), Italian Loneliness Scale (ILS), Multidimensional State Boredom Scale (MSBS), Depression Anxiety Stress Scale (DASS-21) and Toronto Alexithymia Scale (TAS-20).

**Results:**

1,148 respondents (767 women and 374 men, mean age: 23.2±SD=2.8 years old) were recruited. 70.7% declared to have experienced psychological distress. HQ-11 average total score was 18.4±SD=7.5 with statistically significant higher values in the males (p=0.017) and amongst students studying Informatics, Mathematics/Physics/Chemistry, Science of Communication and Engineering. The HQ-11 positively correlated with ILS (r=0.609), MSBS (r=0.415), TAS-20 (r=0.482) and DASS-21 (r=0.434).

**Conclusions:**

This study represents the first screening of the Hikikomori phenomenon in Italian university students. Hikikomori traits appear to be particularly represented in the Italian youth population and should be carefully investigated in future studies.

**Disclosure:**

No significant relationships.

